# Statistical methods for comparing two independent exponential-gamma means with application to single cell protein data

**DOI:** 10.1371/journal.pone.0314705

**Published:** 2024-12-13

**Authors:** Jia Wang, Lili Tian, Li Yan

**Affiliations:** 1 Department of Biostatistics, University at Buffalo, Buffalo, NY, United States of America; 2 Department of Biostatistics and Bioinformatics, Roswell Park Comprehensive Cancer Center, Buffalo, NY, United States of America; Abdul Wali Khan University Mardan, PAKISTAN

## Abstract

In genomic study, log transformation is a common prepossessing step to adjust for skewness in data. This standard approach often assumes that log-transformed data is normally distributed, and two sample t-test (or its modifications) is used for detecting differences between two experimental conditions. However, recently it was shown that two sample t-test can lead to exaggerated false positives, and the Wilcoxon-Mann-Whitney (WMW) test was proposed as an alternative for studies with larger sample sizes. In addition, studies have demonstrated that the specific distribution used in modeling genomic data has profound impact on the interpretation and validity of results. The aim of this paper is three-fold: 1) to present the Exp-gamma distribution (exponential-gamma distribution stands for log-transformed gamma distribution) as a proper biological and statistical model for the analysis of log-transformed protein abundance data from single-cell experiments; 2) to demonstrate the inappropriateness of two sample t-test and the WMW test in analyzing log-transformed protein abundance data; 3) to propose and evaluate statistical inference methods for hypothesis testing and confidence interval estimation when comparing two independent samples under the Exp-gamma distributions. The proposed methods are applied to analyze protein abundance data from a single-cell dataset.

## 1 Introduction

Recent investigations of physical models [1–4] of individual cells have demonstrated that the protein copy number (or abundance) distribution can be approximated by a gamma distribution. These studies claimed that the shape parameter of the gamma distribution can be interpreted as the number of mRNA produced per cell cycle, and the scale parameter as the protein molecules produced per mRNA within individual cells. Although studies at single-cell level were costly and scarce a decade ago, recent technology advances make large scale of protein abundance data at single-cell level proliferate [5, 6].

In practice, up-regulated and down-regulated genes between samples are assessed using fold change which represents a proportional rather than additive changes from a reference (e.g. healthy) to an alternative (e.g. tumor) state. Hence log-transformed abundance level is more biologically relevant, and expression (or concentration) of genes is usually pre-processed using log-transformation before statistical modeling. Additionally, log-transformation is used to adjust for skewness and for variance stabilization [7–11]. Such transformation is widely used as a preprocessing step for many types of molecular markers. Therefore, the exponential-gamma distribution (Exp-gamma), derived from the gamma distribution by applying a logarithmic transformation, is an ideal candidate for modeling log-transformed protein abundance data.

However, researchers often resort to two sample t-test or the Wilcoxon-Mann-Whitney (WMW) test in differential analysis of log-transformed protein abundance and other molecular data [12–14] to detect the difference between two experimental conditions, often with some pre- and post-model adjustment to reduce the false positive rate [15, 16]. Fay and Proschan [17] argued that two sample t-test decision rules are asymptotically valid under quite general conditions even if the normality assumption is rejected. Recently, Li et al. [14] pointed out that two sample t-test often results in exaggerated false positive rate, and recommended using the WMW test for comparing two sets of expression levels measured under two conditions for a gene in population-level RNA-seq studies with large sample sizes. Hao et al. [5] analyzed the differential abundance of cell types across experimental conditions using the WMW test after log-normalization of the protein data. However, some researchers pointed out [17, 18] that although the WMW test does not require parametric assumptions, it assumes that the two distributions are equal under the null hypothesis; hence it could result in inflated type I errors when testing the equality of means. Recently, Torrente et al. [19] studied the shape of gene expression and discovered that the gamma distribution was the predominant non-normal category of genes in both microarray and RNA-seq datasets.

Although there exist some research on the appropriateness of two sample t-test and the WMW test in the differential analysis of log-transformed protein abundance data [5, 12–14], there does not exist such an investigation under the Exp-gamma distribution. Furthermore, accurate statistical inference methods for comparing two Exp-gamma means are of particular interest since identifying differences in log transformed protein abundance data under two different experiment conditions is a fundamental research question in genomics study. Despite the existence of rich statistical research on gamma means [20–29], to our knowledge, there does not exist literature on inference of the Exp-gamma means. Therefore, the aim of this paper is three-fold: 1) to present the Exp-gamma distribution as a proper biological and statistical model for the analysis of log-transformed protein abundance data from single cell experiments; 2) to demonstrate the inappropriateness of using two sample t-test and the WMW test in analyzing log-transformed protein abundance data; 3) to propose and evaluate statistical inference methods for hypothesis testing and confidence interval estimation for comparing two independent samples under Exp-gamma distributions.

This paper is organized as follows. In Section 2, we provide some preliminary results on features of the Exp-gamma distribution, along with its characteristics. In Section 3, the motivation for this research is addressed by a more detailed description of the molecular process of protein production and its critical role in human traits and disease, as given in Section 3.1, followed by an investigation into the inappropriateness of two sample t-test and the WMW test for testing the equality of two Exp-gamma means in Section 3.2. In Section 4, methods for hypothesis testing for the equality of two independent Exp-gamma means and confidence interval estimation for mean difference are proposed. In Section 5, we present the simulation studies on the type I error control and power of the proposed tests, as well as the coverage probability of proposed confidence intervals. In Section 6, a subset of Seurat data used in scRNA-seq studies is analyzed using the proposed methods. Finally, concluding remarks are provided in Section 7.

## 2 The setting

**Fig 1:**
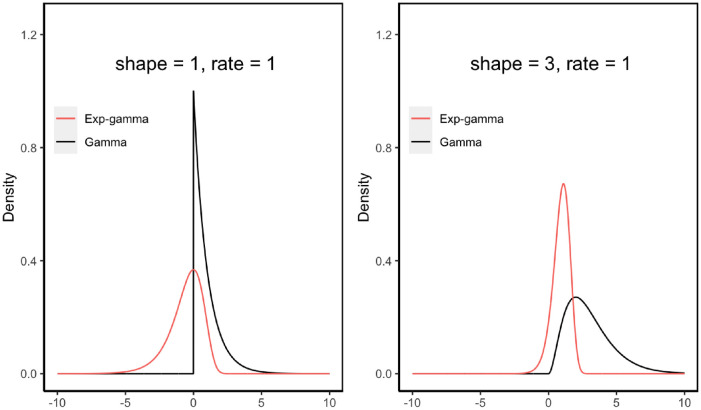
Probability density of *Y* ∼ *Exp-gamma*(*α*, *β*) and *X* = *e*^*Y*^ ∼ *gamma*(*α*, *β*), for (*α*, *β*) = (1, 1) and (3, 1), respectively.

Let *Y*_1_ and *Y*_2_ denote two independent random variables from log-transformed gene expression/protein abundance, where *Y*_*i*_ ∼ *Exp-gamma*(*α*_*i*_, *β*_*i*_), i.e.

$$\begin{array}{c} Y_{i}∼f_{i}\left(y;\alpha_{i},\beta_{i}\right)=\frac{\beta_{i}^{\alpha_{i}}}{\Gamma (\alpha_{i})}e^{\alpha_{i}y}e^{-\beta_{i}e^{y}},\;i=1,2, \end{array}$$

where *y* ∈ (−∞, ∞), and *α*_*i*_, *β*_*i*_ > 0. Note that $X_{i}=e^{Y_{i}}$ following a gamma distribution, i.e. *X*_*i*_ ∼ *gamma*(*α*_*i*_, *β*_*i*_) where *α*_*i*_ and *β*_*i*_ stand for the shape parameter and rate parameter, respectively. Fig 1 contains two graphs of the probability density functions of *Y*_*i*_ and *X*_*i*_ at (*α*_*i*_, *β*_*i*_) = (1, 1) and (*α*_*i*_, *β*_*i*_) = (3, 1), respectively. The Exp-gamma distribution is skewed to the left (negatively skewed), with its both tails extending indefinitely.

Let *δ*_*i*_ and $\sigma_{i}^{2}$ denote the population mean and variance for *Y*_*i*_, respectively. It can be proved that

$$\begin{array}{c} \delta_{i}=\psi \left(\alpha_{i}\right)-\text{ln}\;\beta_{i}, \end{array}$$ (1)

$$\begin{array}{c} \sigma_{i}^{2}=\psi^{\left(1\right)}\left(\alpha_{i}\right), \end{array}$$ (2)

for *i* = 1, 2, where *ψ*() is the digamma function and *ψ*^(1)^() is the trigamma function. The details of the proof are presented in S1 Appendix.

Skewness and excess kurtosis are the other two measures which describe the distributional properties of a probability distribution. Skewness measures the asymmetry of the probability distribution, and excess kurtosis measures how much the distribution deviates from a normal distribution in terms of tails. Both the skewness (*skew*) and the excess kurtosis (*ex-kurt*) of Exp-gamma distribution only depend on its shape parameter *α*_*i*_,

$$\begin{array}{ccc} {\text{skew}}_{i} & = & \psi^{\left(2\right)}\left(\alpha_{i}\right)/{\left[\psi^{\left(1\right)}\left(\alpha_{i}\right)\right]}^{3/2},\\ \text{ex}-{\text{kurt}}_{i} & = & \psi^{\left(3\right)}\left(\alpha_{i}\right)/{\left[\psi^{\left(1\right)}\left(\alpha_{i}\right)\right]}^{2}-3, \end{array}$$ (3)

for *i* = 1, 2, where *ψ*^(*k*−1)^() is *k*th derivative of the log gamma function. The detailed proof is shown in S1 Appendix.

**Fig 2:**
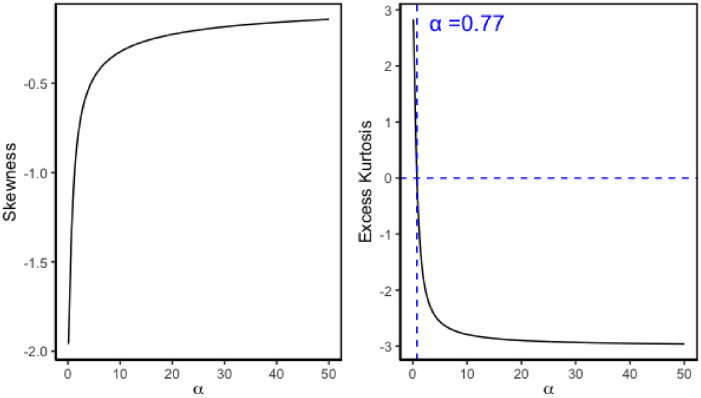
Plot of skewness and excess kurtosis for Exp-gamma distribution as *α* ranges from 0.1 to 50. When *α* = 0.7689, the excess kurtosis is 0.

Fig 2 shows the skewness and excess kurtosis of Exp-gamma distribution as the shape parameter (*α*) ranges from 0.1 to 50. The negative skewness confirms the appearance of Exp-gamma distribution is left skewed. The excess kurtosis of Exp-gamma distribution can be positive and negative, whereas the positive value means that the Exp-gamma distribution is thin-tailed and has fewer outliers, and the negative value means that the Exp-gamma distribution is fat-tailed and has many outliers. When *α* = 0.7689, the Exp-gamma distribution has the same kurtosis as the normal distribution. As *α* tends to infinity, the value of *skew* converges to 0, and the value of *ex-kurt* converges to −3.

We are interested in testing the hypothesis *H*_0_ : *δ*_1_ = *δ*_2_, vs. *H*_1_ : *δ*_1_ ≠ *δ*_2_, as well as constructing confidence interval for mean difference *δ*_1_ − *δ*_2_. The mean difference of two independent Exp-gamma distributions is given by

$$\begin{array}{c} \eta =\delta_{1}-\delta_{2}=\psi \left(\alpha_{1}\right)-\text{ln}\;\beta_{1}-\left(\psi \left(\alpha_{2}\right)-\text{ln}\;\beta_{2}\right). \end{array}$$

Let ${\hat{\alpha }}_{i}$ and ${\hat{\beta }}_{i}$ stand for the maximum likelihood estimates for *α*_*i*_ and *β*_*i*_, respectively. The maximum likelihood estimator (MLE) of *δ*_*i*_ is

$$\begin{array}{c} {\hat{\delta }}_{i}=\psi \left({\hat{\alpha }}_{i}\right)-\text{ln}\;\left({\hat{\beta }}_{i}\right). \end{array}$$

Then

$$\begin{array}{c} \hat{\eta }={\hat{\delta }}_{1}-{\hat{\delta }}_{2}=\psi \left({\hat{\alpha }}_{1}\right)-\text{ln}\;\left({\hat{\beta }}_{1}\right)-\left(\psi \left({\hat{\alpha }}_{2}\right)-\text{ln}\;\left({\hat{\beta }}_{2}\right)\right). \end{array}$$

The variance of $\hat{\eta }$ is

$$\begin{array}{c} \text{Var}\left(\hat{\eta }\right)=\text{Var}\left({\hat{\delta }}_{1}-{\hat{\delta }}_{2}\right)=\frac{\psi^{\left(1\right)}\left({\hat{\alpha }}_{1}\right)}{n_{1}}+\frac{\psi^{\left(1\right)}\left({\hat{\alpha }}_{2}\right)}{n_{2}}. \end{array}$$ (4)

## 3 Motivation

In this section, we provide detailed arguments about the compelling importance of the exponential-gamma (Exp-gamma) distribution in analyzing log-transformed protein abundance data from single-cell experiments, as well as the paramount significance of developing statistical inference procedures under the Exp-gamma distribution.

### 3.1 Justification of using the Exp-gamma distribution for cellular protein abundance measurements

The central dogma of molecular biology is a fundamental theory developed by Francis Crick in 1958 that explains how genetic information flows within a biological system. The core idea can be simply stated as: “DNA makes (messenger) RNA, and RNA makes protein”. The abundance of cellular protein is intimately linked to all biological functions in living cells. Since then, this theory has withstood the test of time and intensive investigations, with only minor exceptions and enrichment. The expression levels of messenger RNAs (mRNAs) and proteins are essential measurements of an organism’s genetic makeup (genotypes), and are often directly related to many observable characteristics or traits (phenotypes), including morphology, development, biochemical, and physiological properties. Common phenotypes in humans include height and blood type, as well as disease related characteristics, e.g. cancer subtypes. Understanding the differences in genotypes (e.g. protein abundance) and their relationships with phenotypes (e.g. cancer progressions) is the focus of molecular biology.

Since its introduction in 2008 [30], cost effective and rapid mRNA quantification of whole genome (transcriptome) has become a standard tool in the life sciences research community. Initially developed for bulk samples, this method evolved to quantify mRNA levels in single cells, and revolutionized the field of cancer research. Numerous analysis methods and pipelines have been developed for mRNA quantification, based on the organism under study, platform characteristics, and researcher’s goals [31]. Due to its wide-spread usage, the mRNA quantification is often used as a synonym for gene expression in many studies. However, in fact, the protein abundance data is a more accurate measurement for gene activity. It is well established that mRNA transcript level only partially correlates with protein abundances [32], and transcriptomics alone is often incapable of distinguishing between categories of cells that are molecularly similar, but functionally distinct. Due to the high cost and experimental complexities, studies that access protein abundance remain scarce, especially at single-cell level because of the low abundance of proteins in cells. Only in the past a few years, genome-wide analysis of protein abundance at single-cell level became practical [5]. Unfortunately, this belated development also means lack of investigation of protein specific statistical analysis method. Most of the methods that were adopted from RNA-seq analysis [7] overlooked sample distribution, except for some preprocessing and normalization steps to compensate for the obvious skewness of the protein data. It becomes clear that single-cell protein abundance specific statistical method for accurate assessment of such data is in great need. Consistent with the two-stage model of gene expression described in the central dogma of molecular biology, the intriguing physical models [1–4] unveiled intrinsic association between gamma distribution parameters and biological process of protein synthesis. Based on these observations, we propose to use the Exp-gamma distribution for modeling single-cell protein levels in molecular biology and cancer research, since log-transformed protein abundances are often biologically more relevant to their cellular functions.

It is worth mentioning that many molecular biology data can be modeled by gamma distribution. For example, microRNA sequencing data often align closely with a gamma distribution due to the stochastic nature of exponential PCR amplification [19, 33, 34]. Additionally, the absolute abundance levels of metabolic [35] and microbiome [36] data exhibit characteristics that align with gamma distributions. Furthermore, log-transformation is a standard preprocessing step in the statistical analysis of these data. Hence, the Exp-gamma distribution is a good candidate for modeling log-transformed cellular protein abundance measurements.

### 3.2 Two sample t-test and the Wilcoxon-Mann-Whitney (WMW) test could be misleading

The two sample t-test and the WMW test are widely used in differential analysis for log-transformed protein abundance data in proteomics [5, 7, 14, 37–39]. However, the appropriateness of using these two tests in differential analysis under the Exp-gamma has not been investigated. Hence, in this section, we aim to use a simulation study to demonstrate their limitations in differential analysis.

Assume two samples of protein abundance obtained under different experimental conditions are from gamma distributions, i.e. *X*_1_ ∼ *gamma*(*α*_1_, *β*_1_), and *X*_2_ ∼ *gamma*(*α*_2_, *β*_2_). The differential analysis is based on log-transformed data from *Y*_1_ and *Y*_2_, where *Y*_1_ = log(*X*_1_) ∼ *Exp-gamma*(*α*_1_, *β*_1_) and *Y*_2_ = log(*X*_2_) ∼ *Exp-gamma*(*α*_2_, *β*_2_). We are interested in testing the equality of two Exp-gamma means.

**Fig 3:**
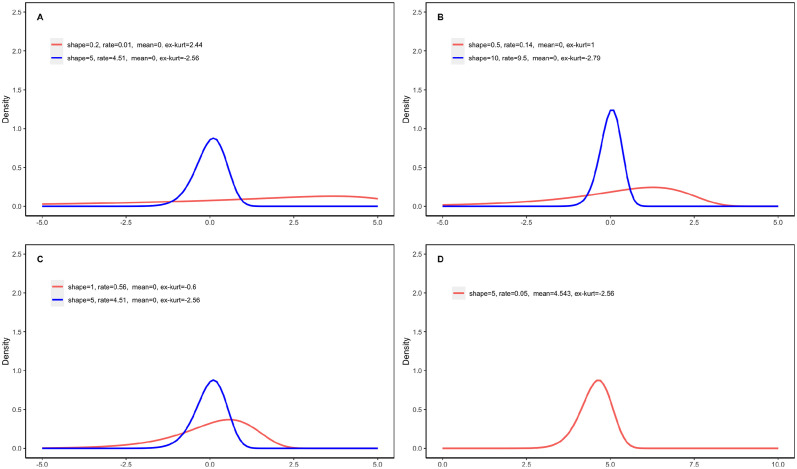
Density plots of samples from four pair of comparisons *Y*_1_ vs *Y*_2_ where *Y*_1_ ∼ *Exp-gamma*(*α*_1_, *β*_1_) vs. *Y*_2_ ∼ *Exp-gamma*(*α*_2_, *β*_2_). (A : (0.2, 0.005) vs. (5, 4.509); B: (0.5, 0.14) vs. (10, 9.504); C: (1, 0.561) vs. (5, 4.509); D: (5, 0.048) vs. (5, 0.048)).

**Fig 4:**
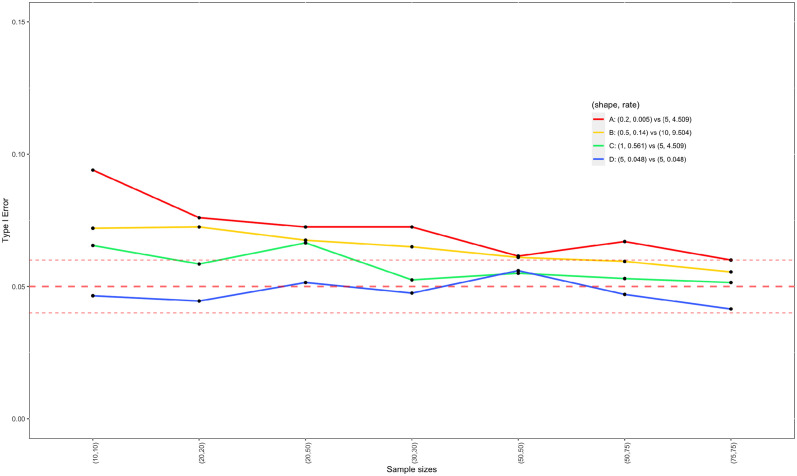
Estimated type I errors of two sample t-test for testing the equality of mean of two Exp-gamma distributions as a function of sample sizes. The middle dashed line represents the nominal significance level at *α* = 0.05; and upper and lower dashed lines are upper and lower limits for satisfactory type I error rates, which are 0.06 and 0.04 with 2000 simulations runs, respectively.

**Fig 5:**
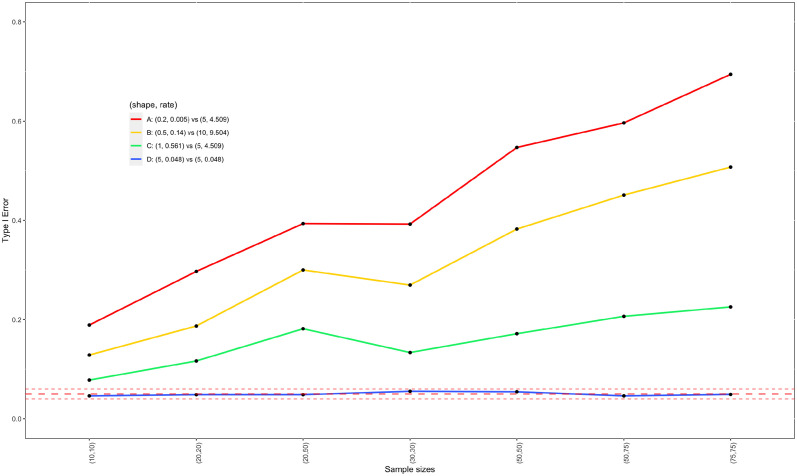
Estimated type I errors of the Wilcoxon-Mann-Whitney (WMW) test for testing the equality of mean of two Exp-gamma distributions as a function of sample sizes. The middle dashed line represents the nominal significance level, which is set to *α* = 0.05; and upper and lower dashed lines are upper and lower limits for the type I error rates, which are 0.06 and 0.04, respectively.

We carried out simulations to evaluate the type I error control of two sample t-test and the WMW test under *H*_0_ : *δ*_1_ − *δ*_2_ = 0. Four parameter settings for (*α*_1_, *β*_1_) vs. (*α*_2_, *β*_2_) are considered: A) (0.2, 0.005) vs. (5, 4.509); B) (0.5, 0.14) vs. (10, 9.504); C) (1, 0.561) vs. (5, 4.509); and D)(5, 0.048) vs. (5, 0.048). In settings A, B, and C, the two Exp-gamma distributions differ, while in setting D, they are identical. Fig 3 presents the density plots under these four settings. It can be seen that these settings vary considerably despite the fact that they are all under *H*_0_ : *δ*_1_ − *δ*_2_ = 0. Under the null hypothesis of equal population means, the probability that *Y*_1_ is greater than *Y*_2_ (i.e. *P*(*Y*_1_ > *Y*_2_)), a measure for the difference between two populations, is 0.621, 0.593, 0.556, and 0.5, for settings A, B, C, and D, respectively, indicating setting A has the largest difference between two populations and setting D has the smallest difference. Note that generally speaking, *P*(*Y*_1_ > *Y*_2_) = 0.5 does not necessarily imply two populations are identical. In this simulation study, we deliberately design setting D to have two identical populations for the purpose of checking the applicability of two sample t-test and the WMW test under two identical Exp-gamma distributions. For each setting, we considered sample sizes from small (10) to large (75). For a given set of sample sizes and parameter configuration, 2000 observed datasets are generated. The simulated type I errors by two sample t-test and the WMW test are reported in Figs 4 and 5, respectively.

As shown in Fig 4, the type I errors of two sample t-test (or Welch’s test for unequal variances) converge to nominal level as sample sizes increase, as guaranteed by the central limit theorem. In addition, when two Exp-gamma distributions are identical (setting D), the two sample t-test maintains controlled type I errors even when sample sizes are small. Note that the type I errors for setting D lie completely between two dashed lines in Fig 4, which indicate boundaries for satisfactory coverage given 2000 simulation runs. However, if two Exp-gamma distributions are different (i.e. settings A, B and C), the type I errors for testing the equality of means can be as high as 0.1, particularly when sample sizes are small (e.g. less than (50, 50) for settings A and B, and less than (30, 30) for setting C). Thus, two sample t-test is appropriate for testing the equality of two means of log-transformed protein abundance data when sample sizes are larger than (50, 50). When dealing with small to medium sample sizes, we should exercise caution with two sample t-test, especially when two underlying distributions are very different.

When the assumption of normality is in doubt, it is a common practice that the WMW test is used as an alternative as it is a non-parametric test. However, while non-parametric tests such as the WMW test do not require normality, they test the null hypothesis that two populations are identical. Hence, when two populations have the same mean but not identical, the WMW test does not guarantee that the significance level will be preserved. More details can be found in the paper by Pratt [18] which thoroughly investigated the effect of differences between two populations on the level of the WMW test for normal, double exponential, and rectangular distributions. In this simulation study, we investigate the effect of the difference between two Exp-gamma distributions on the significance level of the WMW test under null hypothesis of equality of two Exp-gamma means. In Fig 5, we observe inflated type I errors for settings A, B, and C in the WMW test, and the magnitude of inflation increases as sample sizes grow. Furthermore, given sample sizes, as the disparity measured by *P*(*Y*_1_ > *Y*_2_) increases, the inflation of type I error becomes worse; and setting A has the worst type I error control among all settings. It is also notable that the type I errors are well controlled for setting D in which two distributions are identical. Hence, for testing equality of two Exp-gamma means, the WMW test can control type I error only when two distributions are exactly the same, and the type I error can be severely out of control when two distributions are not the same.

In summary, both two sample t-test and the WMW test have limitations in testing of equality of two independent Exp-gamma means. While two sample t-test is not ideal when sample sizes are below medium, the limitations for the WMW test are more severe as it requires the two distributions to be exactly the same under the null hypothesis. In practice, small to medium sizes are common in genomics studies, and scenarios with identical populations under the null hypothesis could be rare. Therefore, accurate procedures for statistical inference for the mean difference of two independent Exp-gamma distributions are desirable.

## 4 Inferences on the mean difference of two independent Exp-gamma distributions

Let *Y*_1_ and *Y*_2_ be two independent Exp-gamma random variables, i.e. *Y*_1_ ∼ *Exp-gamma*(*α*_1_, *β*_1_) and *Y*_2_ ∼ *Exp-gamma*(*α*_2_, *β*_2_). Note that *Y*_*i*_ = ln*X*_*i*_ where *X*_*i*_ ∼ *gamma*(*α*_*i*_, *β*_*i*_), *i* = 1, 2, and *X*_1_ and *X*_2_ are independent. Then the population means for *Y*_1_ and *Y*_2_ are given as follows:

$$\begin{array}{c} \delta_{1}=\psi \left(\alpha_{1}\right)-\text{ln}\;\beta_{1}\;\text{and}\;\delta_{2}=\psi \left(\alpha_{2}\right)-\text{ln}\;\beta_{2}. \end{array}$$

Thus, the research interest is to perform hypothesis testing with satisfactory type I error control under *H*_0_ : *δ*_1_ = *δ*_2_ vs. *H*_1_ : *δ*_1_ ≠ *δ*_2_, and estimate the confidence interval for the mean difference *η* = *δ*_1_ − *δ*_2_ with satisfactory coverage probability. jpeg.

### 4.1 The method based on generalized inference

The concepts of generalized variables and generalized pivots were introduced by Tsui and Weerahandi [40] and Weerahandi [41]. More details can be found in the book of Werrahandi [42]. In S2 Appendix, a brief summary of the core concepts is presented. The concepts of generalized pivotal quantity and generalized confidence interval have been successfully applied to a variety of practical problems when standard exact solutions do not exist, and it has been shown that generalized inference methods generally have good performance, even when sample sizes are small; see e.g. [43–45].

Although there does not exist exact generalized pivots for gamma parameters, approximates generalized pivots have been proposed [22–26]. These approximate pivots have been utilized to make inference for gamma distributions, including single gamma means and difference between two gamma means under different scenarios [27–29]. Utilizing the existing approximate generalized pivots for gamma parameters, we will develop the generalized inference methods for hypothesis testing and confidence interval estimation for mean difference of two independent Exp-gamma distributions.

#### 4.1.1 Generalized pivots for population parameters: A review

Assume *X* ∼ *gamma*(*α*, *β*). In the following, we will first briefly review the existing approximate generalized pivots for gamma parameters *α* and *β*.

Krishnamoorthy and Wang’s method: [25, 26] By applying the Wilson-Hilferty normal approximation, i.e. *W* = *X*^1/3^ ∼ *N*(*μ*, *σ*^2^). Generalized pivotal quantities for normal mean and variance, *R*_*μ*_ and *R*_*σ*^2^_ can be obtained for transformed data. Let $\bar{w}$ and $s_{i}^{2}$ be the observed sample mean and sample variance based on the transformed data *W*. The generalized pivotal quantities for *α* and *β* can be further expressed as:

$$\begin{array}{c} \begin{array}{cc} & R_{\alpha }=\frac{1}{9}\{(1+0.5R_{\mu }^{2}/R_{\sigma^{2}})+{\left[{\left(1+0.5R_{\mu }^{2}/R_{\sigma^{2}}\right)}^{2}-1\right]}^{\frac{1}{2}}\},\;\\ & R_{\beta }=\frac{1}{27{\left(R_{\alpha }\right)}^{\frac{1}{2}}{\left(R_{\sigma^{2}}\right)}^{\frac{3}{2}}}, \end{array} \end{array}$$ (5)

where $R_{\mu }=\bar{w}-\frac{Z}{\sqrt{U_{1}}}\sqrt{\frac{\left(n-1\right)s^{2}}{n}}$, and $R_{\sigma^{2}}=\frac{\left(n-1\right)s^{2}}{U_{2}}∼\frac{\left(n-1\right)s^{2}}{\chi_{n-1}^{2}}$, with *Z* ∼ *N*(0, 1), $U_{1}∼\chi_{n-1}^{2}$, $U_{2}∼\chi_{n-1}^{2}$, and *Z*, *U*_1_, and *U*_2_ are independent.

Chen and Ye’s method: [22, 23] It is known that $2n\alpha \;\text{log}\left(\bar{X}/\tilde{X}\right)∼c\chi_{v}^{2}$ approximately, where *v* = 2*E*^2^(*V*_1_)/Var(*V*_1_) and *c* = *E*(*V*_1_/*v*). The detailed formulas for *E*(*V*_1_) and Var(*V*_1_) can be found in Chen and Ye [22]. Using this result, an approximate generalized pivotal quantity for *α* can be written as

$$\begin{array}{c} R_{\alpha }=V_{1}/\left[2n\;\text{log}(\bar{x}/\tilde{x})\right)], \end{array}$$

where $V_{1}∼\hat{c}\chi_{\hat{v}}^{2}$, $\bar{x}$ and $\tilde{x}$ are observed values of $\bar{X}$ and $\tilde{X}$. Furthermore, utilizing a well-known result regarding gamma distribution, i.e. $2n\beta \bar{X}∼\chi_{2n\alpha }^{2}$, the generalized pivot quantity for *β* can be written as

$$\begin{array}{c} R_{\beta }=V_{2}/\left(2n\bar{x}\right), \end{array}$$ (6)

where $V_{2}∼\chi_{2nR_{\alpha }}^{2}$.

Wang and Wu’s method: [24] Let $T=\text{log}(\tilde{X}/\bar{X})$. Note that *U* = *F*(.) ∼ *U*(0, 1), where *F*(.) is the c.d.f of *T*. On the basis of Cornish-Fisher expansion, the *U*th percentile of *T* can be approximated by *κ*_1_(*α*) + [*κ*_2_(*α*)]^1/2^*Q*(*α*, *U*), where *κ*_*j*_(*α*) is the *j*th cumulant of *T* and *Q*(*α*, *U*) is a function of *κ*_*j*_(*α*)’s. The detailed formulas can be found in Wang and Wu [24]. Let *t* denote the observed value of *T*. An approximate generalized pivotal quantity for *α*, i.e. *R*_*α*_, can be obtained by solving *t* = *κ*_1_(*α*) + [*κ*_2_(*α*)]^1/2^*Q*(*α*, *U*). Similar to Chen and Ye’s method, the approximate generalized pivotal quantity for rate parameter, *R*_*β*_, can be obtained by Eq (6). This method improves Chen and Ye’s method and can work well even when the shape parameter *α* is small.

#### 4.1.2 The generalized inference methods for hypothesis testing and confidence interval estimation for two independent Exp-gamma means

For the *i*th (*i* = 1, 2) sample, the generalized pivotal quantities $R_{\alpha_{i}}$ and $R_{\beta_{i}}$ can be obtained by one of the three approximate generalized inference methods for gamma parameters, i.e. Krishnamoorthy and Wang’s method [25, 26], Chen and Ye’s method [22, 23], and Wang and Wu’s method [24], as reviewed in Section 4.1.1. Replacing *α*_*i*_ with $R_{\alpha_{i}}$ and *β*_*i*_ with $R_{\beta_{i}}$ in Eq (2), the generalized pivotal quantity for *δ*_*i*_ can be expressed as

$$\begin{array}{c} R_{\delta_{i}}=\psi \left(R_{\alpha_{i}}\right)-\text{ln}\;R_{\beta_{i}},\;i=1,2. \end{array}$$ (7)

The generalized pivotal quantity we propose for the mean difference (*η*) of two independent Exp-gamma distributions can be expressed as

$$\begin{array}{c} R_{\eta }=R_{\delta_{1}}-R_{\delta_{2}}=\psi \left(R_{\alpha_{1}}\right)-\text{ln}\;R_{\beta_{1}}-\left(\psi \left(R_{\alpha_{2}}\right)-\text{ln}\;R_{\beta_{2}}\right). \end{array}$$ (8)

It is easy to verify that *R*_*η*_ is a *bona fide* generalized pivotal quantity for *η* approximately. For a given data set $Y_{11},Y_{12},\ldots ,Y_{1n_{1}}$ and $Y_{21},Y_{22},\ldots ,Y_{2n_{2}}$, the following holds: 1) the distribution of *R*_*η*_ is independent of any unknown parameters; 2) the value of *R*_*η*_ is *η* approximately when the statistics used in the definitions of $R_{\alpha_{i}}$ and $R_{\beta_{i}}$ (*i* = 1, 2) are equal their observed value (e.g. in ${\bar{X}}_{i}={\bar{x}}_{i}$ and ${\tilde{X}}_{i}={\tilde{x}}_{i}$ in Chen and Ye’s method).

For testing the hypothesis of equality of two Exp-gamma means,

$$\begin{array}{c} H_{0}:\delta_{1}-\delta_{2}=\eta \;\text{vs.}\;H_{1}:\delta_{1}-\delta_{2}\neq \eta , \end{array}$$ (9)

where *η* = 0. The generalized test variable is defined as

$$\begin{array}{c} T_{\eta }=R_{\eta }-\eta \end{array}$$ (10)

where *R*_*η*_ is the generalized pivotal quantity defined in Eq (8). Note that *T*_*η*_ satisfies the three conditions to be a *bona fide* generalized test variables: 1) the distribution of *T*_*η*_ is free of nuisance parameters; (2) *t*_*η*_, the observed value of *T*_*η*_, is 0, and hence is free of any unknown parameters; and (3) *T*_*η*_ is stochastically decreasing in *η*.

The generalized *p*-value for testing the hypothesis of equality of two Exp-gamma means is given by

$$\begin{array}{c} 2\times \text{min}\{P\left(R_{\eta }\leq 0\right),P\left(R_{\eta }\geq 0\right)\}. \end{array}$$ (11)

#### 4.1.3 Computing algorithm

Consider a given data set *Y*_*ij*_’s (*i* = 1, 2, *j* = 1, 2, …, *n*_*i*_) where the *i*th sample *Y*_*i*_ ∼ *Exp-gamma*(*α*_*i*_, *β*_*i*_). The generalized *p*-value for testing equality of two Exp-gamma means, and estimated confidence interval of the mean difference of two Exp-gamma distributions, can be computed by the following steps:

1. Use one of the three methods presented above, generate $R_{\alpha_{i}}$ and $R_{\beta_{i}}$ for *i* = 1, 2, then compute generalized pivot $R_{\delta_{i}}$ for *δ*_*i*_ following (7) for *i* = 1, 2.
2. Compute generalized pivot $R_{\eta }=R_{\delta_{1}}-R_{\delta_{2}}$ for *η* following (8).
3. Repeat steps 1-2 a total *B* (*B* = 2000) times and obtain array of $R_{\eta }^{b}$’s for *b* = 1, 2, …, *B*.

Let *R*_*η* : *p*_ denote the 100*p* percentile of the *B* *R*_*η*_’s generated in the preceding steps. Then (*R*_*η* : *p*/2_, *R*_*η* : 1 − *p*/2_) is a 100(1 − *p*)% confidence interval for the mean difference of two independent Exp-gamma distributions.

Under the *H*_0_ : *δ*_1_ = *δ*_2_, the generalized *p*-value can be obtained by (11), i.e.

$$\begin{array}{c} p\text{-value}=2\times \text{min}\{\frac{\sum_{i=1}^{B}I_{\left\{R_{\eta }^{b}\leq 0\right\}}}{B},\frac{\sum_{i=1}^{B}I_{\left\{R_{\eta }^{b}\geq 0\right\}}}{B}\}. \end{array}$$ (12)

The *H*_0_ can be rejected if the *p*-value is less than a given significant level *a*.

We refer the three methods based on the generalized pivotal quantity of Exp-gamma mean difference as **G_*K*_**, **G_*C*_**, and **G_*W*_**, corresponding to the methods used for gamma parameters, i.e. Krishnamoorthy and Wang’s method, [25, 26], Chen and Ye’s method [22, 23], and Wang and Wu’s method [24], respectively.

### 4.2 The parametric bootstrap method

Parametric bootstrap (**PB**) method has been widely used in estimating confidence intervals when the parametric model is justified, e.g. [21, 46]. In this section, we propose a **PB** method for hypothesis testing and confidence interval estimation for mean different of two independent Exp-gamma distributions.

Let ${\bar{Y}}_{i}$ denotes the mean based on a sample of size *n*_*i*_ from a *Exp-gamma*(*α*_*i*_, *β*_*i*_) distribution, *i* = 1, 2. Let ${\hat{\alpha }}_{i}$ and ${\hat{\beta }}_{i}$ denote the MLEs of *α*_*i*_ and *β*_*i*_, respectively. Similarly, let ${\bar{Y}}_{i}^{*}$ denotes the mean based on a bootstrap sample of size *n*_*i*_ from the $\text{Exp}-\text{gamma}({\hat{\alpha }}_{i},{\hat{\beta }}_{i})$. Let $\left({\hat{\alpha }}_{i}^{*},{\hat{\beta }}_{i}^{*}\right)$ denote the MLEs based on a boostrap sample, *i* = 1, 2. The **PB** pivot to estimate the difference between two means *δ*_1_ = *ψ*(*α*_1_) − ln(*β*_1_) and *δ*_2_ = *ψ*(*α*_2_) − ln(*β*_2_) is given by

$$\begin{array}{c} Q_{\eta }=\frac{\left({\bar{Y}}_{1}^{*}-{\bar{Y}}_{2}^{*}\right)-\left({\bar{Y}}_{1}-{\bar{Y}}_{2}\right)}{\sqrt{\frac{\psi^{\left(1\right)}\left({\hat{\alpha }}_{1}^{*}\right)}{n_{1}}+\frac{\psi^{\left(1\right)}\left({\hat{\alpha }}_{2}^{*}\right)}{n_{2}}}}. \end{array}$$ (13)

The following steps can be used to obtain the *p*-values for hypothesis testing in Eq (9), decision rules, and confidence interval for *η* based on **PB** method:

1. For a given sample of size *n*_*i*_, calculate the MLEs ${\hat{\alpha }}_{i}$ and ${\hat{\beta }}_{i}$, *i* = 1, 2.
2. Generate bootstrap samples of size *n*_*i*_ from gamma(${\hat{\alpha }}_{i}$, ${\hat{\beta }}_{i}$). Then calculate the ${\bar{Y}}_{i}^{*}$, and MLEs $\left({\hat{\alpha }}_{i}^{*},{\hat{\beta }}_{i}^{*}\right)$ based on the bootstrap samples for *i* = 1, 2.
3. Calculate *Q*_*η*_ as in Eq (13).
4. Repeat steps 2–3 a total *B* (*B* = 2000) times and obtain array of $Q_{\eta }^{b}$’s for *b* = 1, 2, …, *B*.
5. The p-value can be obtained by
  $$\begin{array}{c} p\text{-value}=2\times \text{min}\left\{\frac{\sum_{i=1}^{B}I_{\left\{Q_{\eta }^{b}\leq 0\right\}}}{B},\frac{\sum_{i=1}^{B}I_{\left\{Q_{\eta }^{b}\geq 0\right\}}}{B}\right\},H_{a}:\delta_{1}\neq \delta_{2}. \end{array}$$
6. The *H*_0_ can be rejected if the *p*-values is less than a given significant level *a*.
7. The 100(1 − *p*)% **PB** confidence interval can be obtained as
  $$\begin{array}{c} \{\left({\bar{Y}}_{1}-{\bar{Y}}_{2}\right)-Q_{\eta ;1-p/2}\sqrt{\frac{\psi^{\left(1\right)}\left({\hat{\alpha }}_{1}\right)}{n_{1}}+\frac{\psi^{\left(1\right)}\left({\hat{\alpha }}_{2}\right)}{n_{2}}},\left({\bar{Y}}_{1}-{\bar{Y}}_{2}\right)-Q_{\eta ;p/2}\sqrt{\frac{\psi^{\left(1\right)}\left({\hat{\alpha }}_{1}\right)}{n_{1}}+\frac{\psi^{\left(1\right)}\left({\hat{\alpha }}_{2}\right)}{n_{2}}}\}, \end{array}$$
  where *Q*_*η*;*p*_ denotes the 100*p* percentile of *Q*_*η*_.

## 5 Simulation studies

In previous section, we presented several methods for hypothesis testing and confidence interval estimation for the mean difference between two independent Exp-gamma distributions: three methods based on the generalized pivots (i.e.**G_*C*_**, **G_*W*_**, and **G_*K*_**), and a parametric bootstrap method (i.e. **PB**). Simulation studies are carried out to evaluate the performance of the proposed methods for hypothesis testing and confidence interval estimation.

### 5.1 Hypothesis testing

Sample sizes are set from small (10) to large (75), including both balanced and unbalanced settings. The parameter settings for type I error control include scenarios of equal/unequal shape parameters, with the common mean of two samples ranging from −1.369 to 4.634. The parameter settings for power study include scenarios with equal/unequal shape parameters with the mean difference ranging from 0.5 to 1.386. For each parameter setting, 2000 random samples are generated with given sample sizes. For the type I error and power based on generalized inference methods (**G_*C*_**, **G_*W*_**, and **G_*K*_**), 2000 values of generalized pivots are obtained for each random sample. For the type I error and power obtained by **PB** method, 2000 bootstrap samples are generated for each random sample.

Table 1 presents the type I error rate estimates of hypothesis testing based on the proposed methods (**G_*C*_**, **G_*W*_**, **G_*K*_**, and **PB**), in comparison with t-test and the WMW test, for testing the equality of means of two Exp-gamma distributions. Note that for the first three scenarios, the two Exp-gamma distributions are identical. The remaining scenarios are ranked using *P*(*Y*_1_ > *Y*_2_) in ascending order, indicating a larger disparity between two Exp-gamma distributions under the null hypothesis.

**Table 1:** Estimated type I errors for testing the equality of means of two independent Exp-gamma distributions (2000 simulations).

Scenario	(*α*_1_, *β*_1_) (*α*_2_, *β*_2_)	Mean	*P*(*Y*_1_ > *Y*_2_)	(*skew*_1_, *ex-kurt*_1_)^†^ (*skew*_2_, *ex-kurt*_2_)	Sample size	Type I Error					
						G_*C*_	G_*W*_	G_*K*_	PB	t-test	WMW
1	(1, 1.5) (1, 1.5)	-0.983	0.500	(-1.140, -0.600) (-1.140, -0.600)	(10,10)	0.036	0.057	0.044	0.099	0.058	0.056
					(20,20)	0.038	0.042	0.040	0.066	0.049	0.052
					(20,50)	0.041	0.048	0.036	0.062	0.050	0.048
					(30,30)	0.038	0.042	0.031	0.054	0.039	0.044
					(50,50)	0.042	0.041	0.042	0.052	0.046	0.045
					(50,75)	0.042	0.049	0.043	0.061	0.053	0.047
					(75,75)	0.033	0.037	0.037	0.044	0.038	0.041
2*	(5, 0.048) (5, 0.048)	4.541	0.500	(-0.469, -2.563) (-0.469, -2.563)	(10,10)	0.036	0.035	0.037	0.084	0.047	0.046
					(20,20)	0.039	0.028	0.036	0.057	0.045	0.049
					(20,50)	0.045	0.042	0.042	0.064	0.052	0.049
					(30,30)	0.049	0.043	0.044	0.062	0.048	0.056
					(50,50)	0.057	0.045	0.057	0.057	0.056	0.055
					(50,75)	0.046	0.040	0.044	0.052	0.047	0.046
					(75,75)	0.041	0.037	0.041	0.040	0.042	0.049
3	(50, 0.048) (50, 0.048)	4.634	0.500	(-0.142, -2.960) (-0.142, -2.960)	(10,10)	0.043	0.034	0.040	0.085	0.052	0.048
					(20,20)	0.057	0.047	0.051	0.077	0.057	0.057
					(20,50)	0.045	0.046	0.046	0.057	0.051	0.054
					(30,30)	0.046	0.047	0.049	0.061	0.055	0.055
					(50,50)	0.050	0.045	0.049	0.050	0.047	0.048
					(50,75)	0.052	0.044	0.048	0.054	0.050	0.043
					(75,75)	0.056	0.048	0.055	0.055	0.053	0.056
4	(5, 0.048) (10, 0.101)	4.541	0.512	(-0.469, -2.563) (-0.324, -2.790)	(10,10)	0.028	0.030	0.031	0.073	0.043	0.046
					(20,20)	0.048	0.041	0.045	0.064	0.051	0.058
					(20,50)	0.054	0.051	0.047	0.068	0.057	0.071
					(30,30)	0.044	0.046	0.046	0.061	0.048	0.053
					(50,50)	0.045	0.041	0.047	0.047	0.048	0.051
					(50,75)	0.054	0.056	0.056	0.060	0.056	0.072
					(75,75)	0.053	0.046	0.055	0.051	0.051	0.064
5	(1.5, 4.077) (2, 6)	-1.369	0.513	(-0.917, -1.388) (-0.780, -1.812)	(10,10)	0.022	0.030	0.029	0.078	0.042	0.045
					(20,20)	0.034	0.032	0.033	0.053	0.038	0.045
					(20,50)	0.049	0.044	0.045	0.061	0.052	0.049
					(30,30)	0.045	0.040	0.041	0.058	0.050	0.052
					(50,50)	0.039	0.037	0.042	0.052	0.042	0.052
					(50,75)	0.048	0.038	0.046	0.054	0.047	0.052
					(75,75)	0.044	0.041	0.041	0.054	0.047	0.057
6	(2, 0.200) (4, 0.460)	2.032	0.522	(-0.780, -1.812) (-0.529, -2.443)	(10,10)	0.028	0.034	0.029	0.086	0.046	0.047
					(20,20)	0.040	0.038	0.040	0.057	0.047	0.053
					(20,50)	0.050	0.050	0.041	0.070	0.053	0.087
					(30,30)	0.048	0.044	0.046	0.059	0.050	0.060
					(50,50)	0.050	0.041	0.048	0.054	0.051	0.066
					(50,75)	0.051	0.048	0.054	0.061	0.059	0.087
					(75,75)	0.051	0.046	0.050	0.052	0.049	0.082
7	(2, 0.300) (6, 1.083)	1.627	0.531	(-0.780, -1.812) (-0.425, -2.640)	(10,10)	0.034	0.040	0.038	0.080	0.054	0.054
					(20,20)	0.048	0.047	0.046	0.071	0.053	0.078
					(20,50)	0.050	0.051	0.045	0.078	0.064	0.117
					(30,30)	0.049	0.047	0.050	0.064	0.053	0.089
					(50,50)	0.056	0.049	0.054	0.060	0.058	0.100
					(50,75)	0.048	0.050	0.044	0.052	0.047	0.106
					(75,75)	0.055	0.052	0.055	0.055	0.054	0.124
8	(3, 2.516) (20, 19.502)	0	0.532	(-0.621, -2.237) (-0.226, -2.898)	(10,10)	0.041	0.041	0.037	0.085	0.056	0.065
					(20,20)	0.042	0.037	0.042	0.058	0.046	0.076
					(20,50)	0.048	0.050	0.044	0.069	0.052	0.126
					(30,30)	0.047	0.047	0.046	0.062	0.048	0.082
					(50,50)	0.054	0.058	0.051	0.063	0.057	0.117
					(50,75)	0.055	0.054	0.050	0.061	0.058	0.129
					(75,75)	0.054	0.051	0.062	0.057	0.055	0.116
9	(1, 1.500) (2, 4.077)	-0.983	0.534	(-1.140, -0.600) (-0.780, -1.812)	(10,10)	0.037	0.048	0.038	0.095	0.062	0.059
					(20,20)	0.040	0.043	0.036	0.060	0.047	0.067
					(20,50)	0.046	0.055	0.041	0.075	0.060	0.104
					(30,30)	0.049	0.050	0.051	0.068	0.059	0.085
					(50,50)	0.044	0.048	0.047	0.066	0.052	0.097
					(50,75)	0.047	0.050	0.046	0.057	0.051	0.104
					(75,75)	0.035	0.039	0.044	0.043	0.043	0.114
10	(0.5, 0.14) (1, 0.621)	0	0.553	(-1.535, 1) (-1.140, -0.600)	(10,10)	0.035	0.051	0.035	0.098	0.060	0.069
					(20,20)	0.045	0.059	0.047	0.072	0.060	0.097
					(20,50)	0.035	0.053	0.027	0.060	0.056	0.117
					(30,30)	0.041	0.062	0.056	0.071	0.063	0.125
					(50,50)	0.030	0.051	0.049	0.050	0.045	0.141
					(50,75)	0.034	0.054	0.052	0.059	0.056	0.186
					(75,75)	0.041	0.063	0.086	0.062	0.053	0.218
11	(1, 0.207) (5, 1.659)	1	0.555	(-1.140, -0.600) (-0.469, -2.563)	(10,10)	0.046	0.054	0.044	0.108	0.072	0.090
					(20,20)	0.038	0.046	0.039	0.066	0.055	0.107
					(20,50)	0.043	0.051	0.035	0.068	0.052	0.167
					(30,30)	0.044	0.047	0.041	0.064	0.054	0.133
					(50,50)	0.047	0.056	0.046	0.066	0.059	0.175
					(50,75)	0.044	0.057	0.046	0.057	0.055	0.221
					(75,75)	0.038	0.042	0.048	0.042	0.046	0.246
12*	(1, 0.561) (5, 4.509)	0	0.556	(-1.140, -0.600) (-0.469, -2.563)	(10,10)	0.046	0.050	0.038	0.106	0.066	0.078
					(20,20)	0.044	0.052	0.042	0.079	0.059	0.117
					(20,50)	0.048	0.055	0.041	0.082	0.067	0.182
					(30,30)	0.050	0.056	0.041	0.068	0.053	0.134
					(50,50)	0.043	0.043	0.044	0.056	0.055	0.172
					(50,75)	0.043	0.051	0.041	0.056	0.053	0.207
					(75,75)	0.043	0.045	0.053	0.052	0.052	0.226
13	(1, 0.561) (10, 9.504)	0	0.564	(-1.140, -0.600) (-0.324, -2.790)	(10,10)	0.047	0.052	0.038	0.103	0.063	0.091
					(20,20)	0.036	0.045	0.038	0.068	0.055	0.122
					(20,50)	0.052	0.057	0.042	0.075	0.058	0.213
					(30,30)	0.051	0.052	0.044	0.068	0.060	0.160
					(50,50)	0.042	0.052	0.046	0.057	0.054	0.215
					(50,75)	0.037	0.045	0.040	0.056	0.050	0.243
					(75,75)	0.044	0.050	0.058	0.053	0.052	0.277
14	(1, 0.561) (50, 49.501)	0	0.569	(-1.140, -0.600) (-0.142, -2.960)	(10,10)	0.051	0.053	0.041	0.112	0.061	0.110
					(20,20)	0.051	0.056	0.051	0.082	0.065	0.152
					(20,50)	0.040	0.046	0.032	0.065	0.048	0.222
					(30,30)	0.045	0.049	0.050	0.067	0.059	0.191
					(50,50)	0.040	0.046	0.042	0.055	0.050	0.251
					(50,75)	0.042	0.049	0.042	0.055	0.049	0.288
					(75,75)	0.039	0.045	0.046	0.043	0.046	0.326
15	(0.5, 0.052) (5, 1.659)	1	0.587	(-1.535, 1) (-0.469, -2.563)	(10,10)	0.042	0.057	0.042	0.106	0.073	0.119
					(20,20)	0.045	0.062	0.051	0.072	0.067	0.193
					(20,50)	0.042	0.061	0.042	0.076	0.064	0.299
					(30,30)	0.044	0.055	0.054	0.076	0.068	0.255
					(50,50)	0.042	0.061	0.061	0.067	0.064	0.334
					(50,75)	0.036	0.054	0.065	0.066	0.058	0.409
					(75,75)	0.042	0.057	0.112	0.064	0.061	0.480
16*	(0.5, 0.140) (10, 9.504)	0	0.593	(-1.535, 1) (-0.324, -2.790)	(10,10)	0.040	0.056	0.039	0.113	0.081	0.131
					(20,20)	0.035	0.055	0.048	0.066	0.061	0.211
					(20,50)	0.047	0.053	0.039	0.074	0.068	0.293
					(30,30)	0.043	0.056	0.061	0.078	0.073	0.274
					(50,50)	0.034	0.051	0.061	0.056	0.052	0.390
					(50,75)	0.037	0.055	0.066	0.063	0.058	0.436
					(75,75)	0.044	0.065	0.111	0.068	0.061	0.508
17	(0.5, 0.031) (10, 2.121)	1.500	0.595	(-1.535, 1) (-0.324, -2.790)	(10,10)	0.039	0.056	0.041	0.100	0.074	0.140
					(20,20)	0.039	0.057	0.051	0.071	0.062	0.196
					(20,50)	0.041	0.053	0.034	0.070	0.067	0.299
					(30,30)	0.035	0.047	0.049	0.059	0.058	0.269
					(50,50)	0.029	0.040	0.056	0.053	0.047	0.382
					(50,75)	0.043	0.060	0.074	0.072	0.066	0.441
					(75,75)	0.030	0.042	0.102	0.052	0.049	0.519
18*	(0.2, 0.005) (5, 4.509)	0	0.621	(-1.868, 2.440) (-0.469, -2.563)	(10,10)	0.034	0.054	0.076	0.113	0.094	0.189
					(20,20)	0.032	0.050	0.250	0.074	0.076	0.297
					(20,50)	0.037	0.056	0.174	0.072	0.073	0.394
					(30,30)	0.034	0.057	0.391	0.069	0.073	0.393
					(50,50)	0.035	0.055	0.676	0.059	0.062	0.547
					(50,75)	0.036	0.060	0.667	0.068	0.067	0.597
					(75,75)	0.042	0.059	0.908	0.066	0.060	0.695

* Scenarios discussed in Section 3.2. Scenarios 12, 16, and 18 are the scenarios C, B, and A, respectively.

^†^ *skew*_*i*_ and *ex-kurt*_*i*_ are defined in Eq (3), *i* = 1, 2.

Out of the three proposed methods based on the generalized pivots, **G_*C*_** and **G_*W*_** have excellent type I error control regardless of shapes, rates, sample sizes, and the value of *P*(*Y*_1_ > *Y*_2_). In contract, **G_*K*_** can have inflated type I errors when the shape parameter(s) are small (e.g. scenarios 10, 15–18). The reason is that **G_*K*_** obtains approximate generalized pivotal quantities based on the normal approximation of the distribution with a cube root transformation, and such approximation can be very inaccurate when shape parameter is small [22]. For all scenarios, the **PB** method has inflated type I errors when sample sizes are less than (50, 50). As sample sizes increase, the **PB** method shows improved type I error control. The inflation of type I errors in the **PB** method with small sample sizes is primarily due to the instability in estimating parameters from limited data. Furthermore, this improvement in type I error control does not occur when the shape parameter(s) are small (e.g. scenarios 10, 15–18). Small shape parameters in Exp-gamma distributions lead to highly left-skewed data, which exacerbates the difficulty of accurately estimating the parameters. The type I error of two sample t-test converges to nominal level as sample sizes increase, as guaranteed by the center limit theorem. However, it can have inflated type I errors when sample sizes are less than (50, 50), especially when the disparity between two distributions is obvious (e.g. when *P*(*Y*_1_ > *Y*_2_) is larger than 0.555). For the first three scenarios for which two Exp-gamma distributions are identical, the WMW test has excellent type I error control. However, as the value of *P*(*Y*_1_ > *Y*_2_) deviates from 0.5, the WMW test tends to have more severely inflated type I errors as sample sizes increase. Moreover, the magnitude of type I error inflation increases as the value of *P*(*Y*_1_ > *Y*_2_) becomes larger.

**Fig 6:**
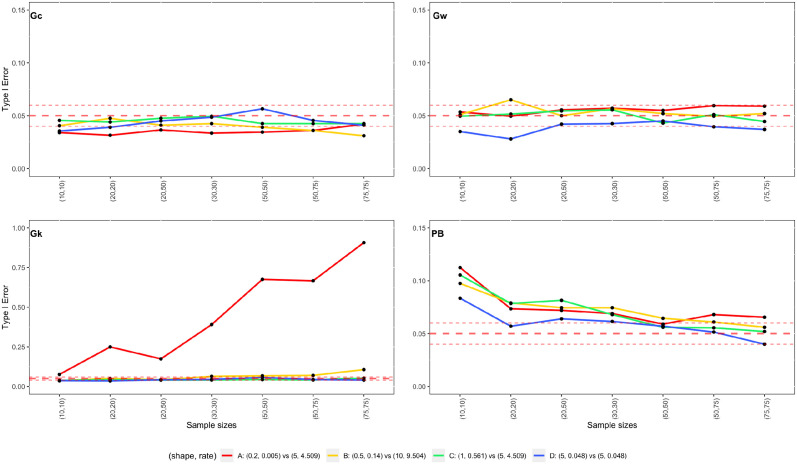
Estimated type I errors of the hypothesis testing based on generalized pivots (G_*C*_, G_*W*_, and G_*K*_) and parametric bootstrap method (PB) for testing the equality of mean of two Exp-gamma distributions as a function of sample sizes. The middle dashed line represents the nominal significance level, which is set to *α* = 0.05; and upper and lower dashed lines are upper and lower limits for the type I error rates, which are 0.06 and 0.04, respectively.

Note that scenarios 2, 12, 16, and 18 in Table 1 are the scenario D, C, B and A, respectively, discussed in Section 3.2 and the type I errors of two sample t-test and the WMW test are presented in Figs 4 and 5. To help to visualize the performance of the proposed methods, Fig 6 presents the type I errors obtained **G_*C*_**, **G_*W*_**, **G_*K*_**, and **PB**, for these four scenarios.

Table 2 presents estimated power of hypothesis testing based on proposed methods (**G_*C*_**, **G_*W*_**, **G_*K*_**, and **PB**), in comparison with t-test and the WMW test.

**Table 2:** Estimated powers for testing the equality of means of two independent Exp-gamma distributions under *H*_1_ : *δ*_1_ ≠ *δ*_2_ (2000 simulations).

Scenario	(*α*_1_, *β*_1_) (*α*_2_, *β*_2_)	*δ*_1_, *δ*_2_ *η* = *δ*_1_ − *δ*_2_	*P*(*Y*_1_ > *Y*_2_)	(*skew*_1_, *ex-kurt*_1_)^†^ (*skew*_2_, *ex-kurt*_2_)	Sample size	Power					
						G_*C*_	G_*W*_	G_*K*_	PB	t-test	WMW
19	(5, 2.735) (0.5, 0.140)	0.500, 0 0.5	0.509	(-0.469, -2.563) (-1.535, 1)	(10,10)	0.134	0.135	0.080	0.144	0.065	0.081
					(20,20)	0.177	0.211	0.093	0.167	0.093	0.081
					(20,50)	0.215	0.243	0.094	0.193	0.109	0.133
					(30,30)	0.242	0.292	0.130	0.234	0.167	0.080
					(50,50)	0.376	0.457	0.180	0.375	0.317	0.086
					(50,75)	0.374	0.454	0.188	0.381	0.316	0.102
					(75,75)	0.517	0.568	0.286	0.500	0.441	0.080
					(75,75)	0.932	0.934	0.924	0.943	0.935	0.856
20	(5, 12.257) (1, 2.516)	-1, -1.5 0.500	0.607	(-0.469, -2.563) (-1.140, -0.600)	(10,10)	0.218	0.226	0.173	0.245	0.138	0.125
					(20,20)	0.434	0.424	0.362	0.417	0.321	0.227
					(20,50)	0.459	0.485	0.390	0.450	0.337	0.305
					(30,30)	0.573	0.561	0.508	0.554	0.479	0.298
					(50,50)	0.779	0.809	0.756	0.786	0.757	0.478
					(50,75)	0.804	0.839	0.774	0.808	0.772	0.538
					(75,75)	0.921	0.935	0.901	0.916	0.902	0.613
21	(5, 1.659) (0.5, 0.14)	1,0 1	0.620	(-0.469, -2.563) (-1.535, 1)	(10,10)	0.376	0.376	0.252	0.363	0.179	0.163
					(20,20)	0.620	0.650	0.487	0.602	0.449	0.264
					(20,50)	0.656	0.681	0.478	0.623	0.467	0.374
					(30,30)	0.777	0.821	0.666	0.769	0.685	0.376
					(50,50)	0.950	0.963	0.880	0.952	0.933	0.560
					(50,75)	0.947	0.966	0.889	0.950	0.933	0.620
					(75,75)	0.995	0.996	0.969	0.990	0.985	0.705
22	(2, 1.5) (1, 1)	0.017, -0.577 0.594	0.640	(-0.469, -2.563) (-1.140,-0.600)	(10,10)	0.171	0.221	0.175	0.285	0.181	0.167
					(20,20)	0.438	0.426	0.408	0.471	0.416	0.349
					(20,50)	0.545	0.549	0.493	0.534	0.443	0.440
					(30,30)	0.603	0.573	0.557	0.618	0.565	0.474
					(50,50)	0.810	0.799	0.810	0.831	0.810	0.696
					(50,75)	0.864	0.870	0.863	0.889	0.858	0.769
					(75,75)	0.932	0.934	0.924	0.943	0.935	0.856
23	(3, 5) (2, 5)	-0.687,-1.187 0.500	0.688	(-0.621, -2.237) (-0.780, -1.812)	(10,10)	0.224	0.261	0.251	0.384	0.290	0.257
					(20,20)	0.562	0.517	0.561	0.616	0.562	0.545
					(20,50)	0.739	0.710	0.714	0.745	0.675	0.695
					(30,30)	0.754	0.720	0.745	0.781	0.758	0.729
					(50,50)	0.931	0.924	0.933	0.939	0.931	0.910
					(50,75)	0.967	0.966	0.968	0.971	0.967	0.952
					(75,75)	0.992	0.987	0.990	0.992	0.990	0.988
24	(2, 5) (2, 10)	-1.187,-1.880 0.693	0.741	(-0.780, -1.812) (-0.780, -1.812)	(10,10)	0.313	0.354	0.353	0.572	0.459	0.439
					(20,20)	0.658	0.691	0.696	0.776	0.749	0.770
					(20,50)	0.752	0.790	0.761	0.885	0.868	0.896
					(30,30)	0.862	0.871	0.873	0.910	0.893	0.906
					(50,50)	0.987	0.984	0.987	0.988	0.989	0.994
					(50,75)	0.994	0.992	0.994	0.996	0.995	0.998
					(75,75)	1.000	1.000	1.000	1.000	1.000	1.000
25	(5, 1.5) (3, 1.5)	1.101,0.517 0.584	0.773	(-0.469, -2.563) (-0.621, -2.237)	(10,10)	0.523	0.540	0.562	0.681	0.591	0.561
					(20,20)	0.912	0.890	0.906	0.932	0.912	0.893
					(20,50)	0.975	0.975	0.972	0.980	0.967	0.964
					(30,30)	0.981	0.975	0.978	0.985	0.981	0.974
					(50,50)	0.999	0.999	0.999	0.999	0.999	0.999
					(50,75)	1.000	1.000	1.000	1.000	1.000	1.000
					(75,75)	1.000	1.000	1.000	1.000	1.000	1.000
26	(3, 5) (3, 10)	-0.687,-1.380 0.693	0.790	(-0.621, -2.237) (-0.621, -2.237)	(10,10)	0.485	0.538	0.530	0.728	0.646	0.617
					(20,20)	0.875	0.892	0.895	0.929	0.917	0.921
					(20,50)	0.947	0.956	0.950	0.975	0.975	0.983
					(30,30)	0.978	0.981	0.982	0.990	0.987	0.987
					(50,50)	1.000	1.000	1.000	1.000	1.000	1.000
					(50,75)	1.000	1.000	1.000	1.000	1.000	1.000
					(75,75)	1.000	1.000	1.000	1.000	1.000	1.000
27	(1, 1) (1, 4)	-0.577,-1.964 1.386	0.800	(-1.140,-0.600) (-1.140,-0.600)	(10,10)	0.375	0.490	0.493	0.716	0.646	0.669
					(20,20)	0.804	0.869	0.884	0.926	0.914	0.952
					(20,50)	0.874	0.913	0.907	0.951	0.953	0.979
					(30,30)	0.954	0.971	0.977	0.983	0.981	0.993
					(50,50)	0.998	1.000	1.000	1.000	1.000	1.000
					(50,75)	0.999	1.000	1.000	1.000	1.000	1.000
					(75,75)	1.000	1.000	1.000	1.000	1.000	1.000

^†^ *skew*_*i*_ and *ex-kurt*_*i*_ are defined in Eq (3), *i* = 1, 2.

Reflecting on the type I error control presented in Table 1, caution should be exercised while interpreting estimated power and making comparisons between methods. Note the following: 1) the power of the WMW method when the value of *P*(*Y*_1_ > *Y*_2_) deviates from 0.5 is not interpretable due to its inflated type I error for these cases, 2) the power of two sample t-test might be inflated when sample sizes less than 50 due to its inflated type I error for these cases; 3) the power of the PB method could be inflated due to its poor type I error control, especially at small sample sizes; 4) **G_*K*_** can have inflated power due to inflated type I error when the shape parameter (*α*) is small. For example, for scenarios 26 and 27 where the value of *P*(*Y*_1_ > *Y*_2_) has a larger deviation from 0.5, the WMW test and two sample t-test have higher power than that of **G_*C*_**, **G_*W*_** and **G_*K*_** when sample sizes are small. Such observations are due to the inflated type I error for the WMW test and the two sample t-test; hence they should not be interpreted as an evidence that t-test and the WMW test are more powerful.

Overall speaking, the two generalized inference methods with good type I error control, i.e. **G_*C*_** and **G_*W*_**, have comparable power. When sample sizes exceed (50, 50), the powers by two sample t-test and the PB test are comparable to those of **G_*C*_** and **G_*W*_**.

In summary, we recommend both **G_*C*_** and **G_*W*_** methods for hypothesis testing of two independent Exp-gamma distributions due to their ability to provide decent power with excellent type I error control, even when sample sizes are small. The **G_*K*_** method is not recommended because it has inflated type I errors for certain scenarios, such as when the shape parameter is less than 0.5. The **PB** method has inflated type I errors when sample sizes are small or when shape parameter(s) are small, leading to incorrect rejection of the null hypothesis. Two sample t-test may exhibit inflated type I errors at small sample sizes. The WMW test only maintains controlled type I errors when the two distributions are identical, hence it is not a reliable choice.

### 5.2 Confidence intervals

The proposed three methods based on the generalized pivots (i.e. **G_*C*_**, **G_*W*_**, and **G_*K*_**), and parametric bootstrap (**PB**) method can provide estimated confidence interval for the mean difference between two Exp-gamma distributions. Additionally, the estimated confidence intervals by two sample t-test are also provided for comparison purpose. Note that theoretically, the WMW method can not yield estimated confidence interval for the mean difference.

Simulation studies are carried out to evaluate the performances of proposed methods regarding coverage probabilities and the average lengths of proposed confidence intervals for mean difference of two independent Exp-gamma distributions. The sample sizes are set as (10, 10), (20, 20), (30, 30), (20, 50), (50, 50), (50, 75), and (75, 75). We considered settings with equal means (i.e. *η* = 0), as well as different means (i.e. *η* ≠ 0), and with equal/unequal shape parameters. For each parameter setting, 2000 samples are simulated. For generalized confidence intervals, 2000 *R*_*η*_’s are obtained. For **PB** method, *B* = 2000 bootstrap samples are used.

Table 3 presents the coverage probabilities and average lengths of proposed confidence intervals. Overall speaking, the **G_*C*_** and **G_*W*_** methods that based on the generalized pivots maintain satisfactory coverage probabilities for all settings except that they might be slightly conservative at small sample sizes such as (10, 10), while the **G_*K*_** method is not recommended when the shape parameter is less than 0.5, due to the fact that this normal-based method does not work well when shape parameter is small [22]. The confidence intervals obtained by the **PB** method are liberal when sample sizes are small, although its coverage probabilities converge to nominal level when sample sizes reach (50, 50). The coverage probabilities of the two sample t-test converges to nominal level as sample sizes increase. However, for some scenarios, it can be liberal when sample sizes are small, such as scenario 19 as sample sizes being less than (30, 30), and scenario 20 at (10, 10). In terms of the length of confidence intervals, the **PB** method appears to provide shortest confidence intervals among the proposed methods when sample sizes are small. However, this observation is due to the fact that the **PB** method is liberal at small sizes, hence it should not be interpreted. As sample sizes reach (50, 50), all four methods are generally comparable in terms of length.

**Table 3:** Coverage probabilities and average lengths of proposed 95% confidence intervals^†^ for mean difference of two independent Exp-gamma distributions (2000 simulations).

Scenario*	(*α*_1_, *β*_1_) (*α*_2_, *β*_2_)	*η*	Sample size	Coverage probability(Average length)				
				G_*C*_	G_*W*_	G_*K*_	PB	t-test
2	(5, 0.048) (5, 0.048)	0	(10,10)	0.965 (1.021)	0.965 (0.994)	0.963 (0.970)	0.916 (0.773)	0.954 (0.872)
			(20,20)	0.962 (0.627)	0.972 (0.652)	0.965 (0.628)	0.943 (0.569)	0.956 (0.599)
			(20,50)	0.955 (0.523)	0.958 (0.531)	0.958 (0.527)	0.936 (0.476)	0.949 (0.499)
			(30,30)	0.952 (0.496)	0.958 (0.517)	0.956 (0.503)	0.939 (0.468)	0.953 (0.485)
			(50,50)	0.944 (0.375)	0.955 (0.391)	0.943 (0.375)	0.943 (0.370)	0.944 (0.373)
			(50,75)	0.955 (0.341)	0.961 (0.353)	0.957 (0.341)	0.949 (0.335)	0.953 (0.339)
			(75,75)	0.959 (0.304)	0.963 (0.313)	0.960 (0.305)	0.960 (0.303)	0.959 (0.303)
3	(50, 0.481) (50, 0.481)	0	(10,10)	0.958 (0.282)	0.966 (0.296)	0.960 (0.284)	0.915 (0.234)	0.949 (0.265)
			(20,20)	0.944 (0.184)	0.954 (0.194)	0.949 (0.188)	0.923 (0.171)	0.943 (0.181)
			(20,50)	0.956 (0.158)	0.955 (0.157)	0.955 (0.157)	0.943 (0.145)	0.949 (0.152)
			(30,30)	0.954 (0.151)	0.954 (0.153)	0.952 (0.151)	0.940 (0.142)	0.945 (0.146)
			(50,50)	0.951 (0.112)	0.955 (0.115)	0.951 (0.113)	0.950 (0.112)	0.953 (0.113)
			(50,75)	0.949 (0.102)	0.956 (0.105)	0.952 (0.103)	0.947 (0.102)	0.951 (0.103)
			(75,75)	0.945 (0.091)	0.952 (0.093)	0.946 (0.091)	0.945 (0.092)	0.948 (0.092)
4	(5, 0.048) (10, 0.101)	0	(10,10)	0.973 (0.864)	0.971 (0.850)	0.970 (0.841)	0.927 (0.664)	0.957 (0.756)
			(20,20)	0.953 (0.536)	0.959 (0.554)	0.955 (0.539)	0.936 (0.490)	0.949 (0.516)
			(20,50)	0.946 (0.481)	0.950 (0.486)	0.954 (0.493)	0.932 (0.439)	0.943 (0.465)
			(30,30)	0.956 (0.435)	0.954 (0.441)	0.954 (0.430)	0.940 (0.404)	0.952 (0.418)
			(50,50)	0.956 (0.323)	0.959 (0.330)	0.953 (0.321)	0.953 (0.318)	0.953 (0.321)
			(50,75)	0.947 (0.307)	0.945 (0.308)	0.944 (0.305)	0.941 (0.298)	0.944 (0.302)
			(75,75)	0.948 (0.259)	0.954 (0.265)	0.945 (0.259)	0.949 (0.262)	0.950 (0.261)
1–96	(2, 0.200) (4, 0.460)	0	(10,10)	0.973 (1.607)	0.967 (1.457)	0.972 (1.474)	0.914 (1.108)	0.955 (1.271)
			(20,20)	0.961 (0.953)	0.963 (0.948)	0.961 (0.925)	0.943 (0.822)	0.954 (0.872)
			(20,50)	0.951 (0.852)	0.951 (0.841)	0.960 (0.857)	0.930 (0.743)	0.948 (0.792)
			(30,30)	0.952 (0.737)	0.957 (0.744)	0.954 (0.727)	0.941 (0.675)	0.951 (0.700)
			(50,50)	0.951 (0.544)	0.959 (0.563)	0.953 (0.546)	0.947 (0.538)	0.949 (0.540)
			(50,75)	0.949 (0.523)	0.953 (0.532)	0.947 (0.520)	0.939 (0.509)	0.942 (0.512)
			(75,75)	0.950 (0.440)	0.955 (0.455)	0.950 (0.438)	0.949 (0.438)	0.951 (0.440)
7	(2, 0.300) (6, 1.083)	0	(10,10)	0.967 (1.521)	0.961 (1.387)	0.963 (1.414)	0.920 (1.055)	0.947 (1.219)
			(20,20)	0.953 (0.897)	0.954 (0.887)	0.955 (0.871)	0.929 (0.772)	0.947 (0.823)
			(20,50)	0.950 (0.827)	0.950 (0.817)	0.956 (0.834)	0.922 (0.720)	0.936 (0.769)
			(30,30)	0.951 (0.699)	0.953 (0.705)	0.951 (0.690)	0.936 (0.641)	0.948 (0.664)
			(50,50)	0.944 (0.515)	0.951 (0.529)	0.947 (0.517)	0.940 (0.506)	0.942 (0.509)
			(50,75)	0.952 (0.502)	0.951 (0.502)	0.956 (0.502)	0.948 (0.489)	0.953 (0.491)
			(75,75)	0.946 (0.413)	0.949 (0.424)	0.945 (0.411)	0.945 (0.413)	0.947 (0.415)
19	(5, 2.735) (0.5, 0.140)	0.5	(10,10)	0.955 (4.470)	0.944 (3.624)	0.961 (3.282)	0.880 (2.580)	0.916 (3.020)
			(20,20)	0.962 (2.458)	0.939 (2.172)	0.941 (1.975)	0.920 (1.936)	0.928 (2.044)
			(20,50)	0.955 (2.407)	0.942 (2.150)	0.967 (2.083)	0.931 (1.920)	0.940 (2.028)
			(30,30)	0.970 (1.932)	0.952 (1.711)	0.949 (1.601)	0.938 (1.592)	0.936 (1.657)
			(50,50)	0.968 (1.443)	0.949 (1.291)	0.948 (1.238)	0.941 (1.230)	0.949 (1.280)
			(50,75)	0.968 (1.415)	0.944 (1.288)	0.931 (1.205)	0.934 (1.211)	0.944 (1.262)
			(75,75)	0.961 (1.101)	0.951 (1.034)	0.894 (0.942)	0.944 (1.010)	0.949 (1.029)
20	(5, 12.257) (1, 2.516)	0.5	(10,10)	0.955 (2.472)	0.947 (2.084)	0.956 (2.091)	0.891 (1.545)	0.928 (1.796)
			(20,20)	0.963 (1.401)	0.954 (1.318)	0.961 (1.302)	0.933 (1.160)	0.946 (1.234)
			(20,50)	0.958 (1.341)	0.949 (1.258)	0.965 (1.307)	0.932 (1.120)	0.948 (1.199)
			(30,30)	0.957 (1.083)	0.953 (1.043)	0.959 (1.039)	0.935 (0.952)	0.946 (0.997)
			(50,50)	0.953 (0.816)	0.944 (0.786)	0.954 (0.787)	0.935 (0.743)	0.942 (0.764)
			(50,75)	0.957 (0.805)	0.944 (0.767)	0.955 (0.775)	0.941 (0.730)	0.946 (0.752)
			(75,75)	0.962 (0.634)	0.959 (0.630)	0.952 (0.607)	0.958 (0.615)	0.954 (0.620)
25	(5, 1.5) (3, 1.5)	0.583	(10,10)	0.957 (1.243)	0.957 (1.174)	0.956 (1.168)	0.906 (0.906)	0.942 (1.030)
			(20,20)	0.956 (0.756)	0.963 (0.769)	0.960 (0.747)	0.940 (0.670)	0.951 (0.709)
			(20,50)	0.961 (0.667)	0.961 (0.669)	0.963 (0.673)	0.934 (0.594)	0.949 (0.629)
			(30,30)	0.950 (0.593)	0.958 (0.610)	0.951 (0.594)	0.932 (0.553)	0.944 (0.574)
			(50,50)	0.948 (0.438)	0.959 (0.459)	0.949 (0.441)	0.947 (0.435)	0.950 (0.438)
			(50,75)	0.951 (0.420)	0.950 (0.427)	0.950 (0.417)	0.948 (0.409)	0.949 (0.414)
			(75,75)	0.943 (0.355)	0.952 (0.371)	0.941 (0.357)	0.944 (0.356)	0.944 (0.357)
26	(3, 5) (3, 10)	0.693	(10,10)	0.966 (1.459)	0.963 (1.356)	0.962 (1.332)	0.923 (1.033)	0.953 (1.166)
			(20,20)	0.954 (0.862)	0.961 (0.873)	0.957 (0.846)	0.932 (0.759)	0.952 (0.800)
			(20,50)	0.958 (0.706)	0.960 (0.707)	0.961 (0.710)	0.941 (0.634)	0.955 (0.665)
			(30,30)	0.953 (0.675)	0.964 (0.692)	0.958 (0.675)	0.936 (0.622)	0.952 (0.644)
			(50,50)	0.946 (0.500)	0.960 (0.523)	0.948 (0.503)	0.943 (0.489)	0.949 (0.497)
			(50,75)	0.950 (0.455)	0.958 (0.474)	0.953 (0.458)	0.948 (0.448)	0.955 (0.454)
			(75,75)	0.942 (0.402)	0.954 (0.421)	0.949 (0.407)	0.940 (0.398)	0.949 (0.404)

* Scenarios in Table 3 are subset of scenarios in Tables 1 and 2.

^†^ The confidence interval for the WMW test is not applicable.

In summary, generally we recommend the proposed **G_*C*_** and **G_*W*_** methods over **G_*K*_**, **PB** method, and two sample t-test, due to the fact that **G_*C*_** and **G_*W*_** methods maintain satisfactory coverage probabilities even at small sample sizes and when the shape parameters are small.

## 6 Data examples

In this section, we illustrate the proposed method using publicly accessible data from a recent study that measured mRNA expression and protein abundance at single cell level simultaneously by Hao et.al. [5]. In this study, peripheral blood mononuclear cell (PBMC) samples from eight volunteers were collected at pre (day 0) and post HIV vaccination (day 3 and 7), yielding a total of 210,911 cells. The CITE-seq method was used to simultaneously quantify RNA and surface protein abundance in single cells via the sequencing of antibody-derived tags (ADTs). Analyses identified 57 clusters of different types of cells, encapsulated all major and minor immune cell types and revealed striking cellular diversity.

For demonstration purpose without delving deeply into the biological details of immune cells functions, we focus on protein abundance data in the cluster of plasmacytoid dendritic Cell (pDC) cells. The pDC releases type 1 interferon in response to viral infection [47], thus could serve as an indicator of immune response to vaccination. Although pDC cell counts are usually low in PBMC samples, as shown in Hao’s study, they may play a critical role in regulating gene expression and innate immune responses [48]. The data used in this manuscript were attached as S1 Table. Based on the physical model [1–4], it is reasonable to assume the measured protein abundances of individual cells follow Gamma distribution. This assumption is further verified by goodness-of-fit test [49] for Gamma distribution using method implemented in R goft package. The biological effects were estimated by comparing the log-transformed protein abundances.

In this section, different analyses are performed to investigate the protein abundance variation between donors and across time points within pDC cells. According to the simulation results in Section 5, the **PB** method is not suitable, as it yields inflated type I errors when sample sizes are small, which is very common for protein abundance data. Furthermore, **G_*K*_**, one of the methods based on generalized pivots, generates inaccurate results when the shape parameter is small, potentially leading to unreliable testing outcomes. Thus, we use two recommended testing approaches based on the generalized pivots (i.e.**G_*C*_** and **G_*W*_**) on the log-transformed data. For comparison purpose, we also analyze data using two sample t-test and the WMW test, which are commonly used in the differential analysis of protein abundance data and other genomic studies. More details described in **Example 1** and **Example 2** below. To enable direct comparisons between different donors regardless of differences in sample size, we use relative counts (RC) of protein abundance. In the settings of this single-cell studies, the sample sizes refer to the counts of pDC cells, making our proposed methods ideal choices for modeling them.

**Example 1.** Comparison of log-transformed protein abundance data for two different donors at same time point.

Analyzing protein abundance data across different donors at given time points allows us to perceive variations in the immune response to vaccination among different individuals. Since the consistency of immune response is crucial for vaccine success, accurately assessing protein levels at fixed time related to vaccination is essential for evaluating its quality and effectiveness.

Table 4 lists summary statistics for four genes: Rat-IgG1–2 (donor P1 vs. P3 at day 7), CD3–2 (donor P3 vs. P8 at day 0), CD226 (donor P1 vs. P6 at day 0), and CD44–2 (donor P1 vs. P3 at day 3). The estimated *p*-values as well as confidence intervals by the proposed methods (**G_*C*_** and **G_*W*_**) and two sample t-test and the WMW test are also presented. For these four proteins, the **G_*C*_** and **G_*W*_** methods yield different conclusions in terms of significance, in contrast to two sample t-test and the WMW test.

**Table 4:** Testing the equality of protein abundance data from different donors at the same time point (*p*-value and estimated confidence interval for mean difference).

Protein	Time	Donor 1 Donor 2	*n*_1_ *n*_2_	(${\hat{\alpha }}_{1}$, ${\hat{\beta }}_{1}$) (${\hat{\alpha }}_{2}$, ${\hat{\beta }}_{2}$)	${\hat{\delta }}_{1}$ ${\hat{\delta }}_{2}$	$\hat{\eta }$	Methods	*p*-value	Est. CI* (lower, upper)
Rat-IgG1–2	7	P1	22	(9.800, 0.366)	3.236	0.339	G_*C*_	0.039	(0.020, 0.717)
		P3	17	(3.440, 0.163)	2.897		G_*W*_	0.033	(0.037, 0.719)
							t-test	0.055	(-0.007, 0.684)
							WMW	0.092	^†^
CD38–2	0	P3	19	(5.520, 0.075)	4.212	0.524	G_*C*_	0.027	(0.062, 1.290)
		P8	12	(2.220, 0.044)	3.688		G_*W*_	0.034	(0.061, 1.190)
							t-test	0.051	(-0.003, 1.060)
							WMW	0.064	^†^
CD226	0	P1	27	(5.240, 0.190)	3.219	-0.379	G_*C*_	0.075	(-0.737, 0.038)
		P6	17	(3.130, 0.072)	3.598		G_*W*_	0.065	(-0.719, 0.021)
							t-test	0.046	(-0.755, -0.007)
							WMW	0.018	^†^
CD44–2	3	P1	8	(9.410, 0.104)	4.451	0.324	G_*C*_	0.069	(-0.034, 0.606)
		P3	42	(6.641, 0.099)	4.127		G_*W*_	0.067	(-0.038, 0.620)
							t-test	0.041	(0.016, 0.637)
							WMW	0.044	^†^

* Estimated confidence interval.

^†^ The Est. CI for the WMW test is not applicable.

As observed in simulation studies, the two sample t-test is unreliable as the disparity between two distributions is large, especially when sample sizes are less than (50, 50). Moreover, the WMW test is very sensitive to difference between the shapes of distributions. For this data set, the sample sizes (counts of pDC cells) are generally small, and the data exhibit different distributions for different donors at same time point. Therefore, two sample t-test and the WMW test should not be trusted for their testing results.

For instance, when comparing the protein abundance of Rat-IgG1–2 between donor P1 and donor P3 at day 7, our proposed approaches (**G_*C*_** and **G_*W*_**) reveal significant mean difference. However, two sample t-test and the WMW test fail to identify this difference. On the other hand, when examining the protein abundance of CD226 between donor P1 and donor P6 at day 0, our **G_*C*_** and **G_*W*_** methods indicate there are no significance between two donors, whereas two sample t-test and the WMW test state otherwise. These erroneous conclusions based on t-test and the WMW test may lead us to mis-characterize the nature of vaccine response related to these genes.

Furthermore, the estimated 95% confidence intervals for the mean difference (*η*) are also presented in Table 4, and **G_*C*_** and **G_*K*_** methods generally have the comparable lengths.

**Example 2.** Comparison of log-transformed protein abundance data at two different times for same donor.

One important aspect of the study by Hao et al. [5] is to characterize the response to vaccination for each of previously identified cell types, with particular interests in identifying cell populations that contribute most strongly to the innate immune response. This response is expected to be highly activated at the first vaccinated time point (day 3), and subsequently dampen at the second time point (day 7), as observed with another non-replicating viral vectored HIV vaccine [50]. Therefore, donor with strong innate immune response to vaccination and the activated genes can be identified by comparing the gene expression at time 0 to 3 and 7, as described in Hao et al. [5] The ability to identification individual with strong innate immune response is critical in our understanding of antibody production related to vaccine and other factors. Such insight may also help us developing more personalized treatment. Finally, we would like to point out that, due to the nature of single cell experiments, the gene protein levels at different time points are extracted from independent sets of cells. Therefore, t-test and WMW test of two independent samples were used when we investigate the changes of means of log-transformed protein abundance between two times for a given donor in this example.

Table 5 lists summary statistics for three genes (CD48, CD45–1, and CD337) of donor P8 for day 0 vs. day 3, and day 0 vs. day 7. The estimated *p*-values as well as confidence intervals by the proposed methods (**G_*C*_** and **G_*W*_**), and the commonly used two sample t-test and the WMW test, are presented. For these three genes, the proposed methods may or may not yield different conclusions in terms of significance, comparing to the two sample t-test and the WMW test.

**Table 5:** Testing the equality of protein abundance data from same donor at different time points (*p*-value and estimated confidence interval for mean difference).

Protein	Donor	Time 1 Time 2	*n*_1_ *n*_2_	(${\hat{\alpha }}_{1}$, ${\hat{\beta }}_{1}$) (${\hat{\alpha }}_{2}$, ${\hat{\beta }}_{2}$)	${\hat{\delta }}_{1}$ ${\hat{\delta }}_{2}$	$\hat{\eta }$	Methods	*p*-value	Est. CI* (lower, upper)
CD48	P8	0	12	(13.700, 0.080)	5.112	-0.190	G_*C*_	0.045	(-0.411, -0.005)
		3	34	(17.700, 0.086)	5.302		G_*W*_	0.042	(-0.413, -0.006)
							t-test	0.054	(-0.392, 0.004)
							WMW	0.097	^†^
CD48	P8	0	12	(13.700, 0.080)	5.112	0.040	G_*C*_	0.761	(-0.207, 0.281)
		7	20	(9.750, 0.058)	5.072		G_*W*_	0.702	(-0.195, 0.268)
							t-test	0.710	(-0.187, 0.270)
							WMW	0.526	^†^
CD45–1	P8	0	12	(0.730, 0.174)	0.611	-0.914	G_*C*_	0.049	(-3.100, -0.005)
		3	34	(1.610, 0.249)	1.525		G_*W*_	0.037	(-2.460, -0.071)
							t-test	0.124	(-2.120, 0.286)
							WMW	0.101	^†^
CD45–1	P8	0	12	(0.730, 0.174)	0.611	0.139	G_*C*_	0.923	(-2.150, 1.800)
		7	20	(0.615, 0.141)	0.472		G_*W*_	0.900	(-1.520, 1.480)
							t-test	0.832	(-1.260, 1.550)
							WMW	0.953	^†^
CD337	P8	0	12	(3.190, 0.608)	1.493	0.860	G_*C*_	0.026	(0.108, 1.870)
		3	34	(0.648, 0.134)	0.633		G_*W*_	0.028	(0.135, 1.670)
							t-test	0.023	(0.124, 1.590)
							WMW	0.506	^†^
CD337	P8	0	12	(3.190, 0.608)	1.493	0.127	G_*C*_	0.699	(-0.516, 0.831)
		7	20	(1.510, 0.267)	1.366		G_*W*_	0.689	(-0.493, 0.788)
							t-test	0.685	(-0.489, 0.735)
							WMW	0.833	^†^

* Estimated confidence interval.

^†^ The Est. CI for the WMW test is not applicable.

For example, when comparing the protein abundance of CD48 between day 0 and 3 for donor P8, our proposed approaches (**G_*C*_** and **G_*W*_**) identify significant mean difference in log-transformed samples. Furthermore, the immune response is dampens at day 7, and the **G_*C*_** and **G_*W*_** methods yield insignificant difference from day 0 to 7. This pattern aligns with the characteristics of innate immune response stated above. However, both two sample t-test and the WMW test fail to generate significant differences between day 0 and day 3, indicating that the changes of CD48 abundance do not fit the pattern. Similar patterns are observed for CD337 and CD45–1 by the **G_*C*_** and **G_*W*_** methods, while such discoveries would have been missed by either the WMW test or t-test.

Interestingly, genes CD48 [51], CD45–1 [52] and CD337 [48] are all playing important roles in human’s immune system. The observed multiple protein abundance modifications in donor P8 may indicate a different innate immune response compared to other donors, which do not show the patterns of changes described above. This discovery may point to potential existence of minority subtypes with different responses to the vaccine. A closer examination of changes in immune-related gene profiles in response to the vaccine might have clinical value.

Furthermore, the estimated 95% confidence intervals for the mean difference (*η*) by proposed methods are presented in Table 5.

## 7 Summary and discussion

In genomics, the two sample t-test and the Wilcoxon-Mann-Whitney (WMW) test are commonly used to identify proteins that can differentiate between different experiment conditions, and the comparison is usually applied on log-transformed protein abundance data [39]. However, the protein abundance data could be modeled by gamma distribution [3, 53, 54], and the shape of protein abundance distribution needs to be taken into consideration in differential analysis [19].

In this paper, we demonstrated the inappropriateness of using two sample t-test and the WMW test for testing the equality of means of two log-transformed protein abundance samples. Several methods for two-sample hypothesis testing and confidence interval estimation for mean difference of two independent Exp-gamma distributions are proposed.

Through comprehensive simulation studies, we demonstrated that two proposed methods (i.e. **G_*C*_** and **G_*W*_**) based on the concepts of generalized inference can have excellent type I error control for testing the equality of two Exp-gamma means. Additionally, these methods can provide satisfactory confidence intervals for the Exp-gamma mean difference, with consistent performance across different parameter settings and sample sizes. Furthermore, the **G_*C*_** and **G_*W*_** methods work well even when the data are highly skewed. On the other hands, the **G_*K*_** method is not recommended when the shape parameter(s) are less than 0.5, as the cube root transformation is not accurate with small shape parameter(s). The **PB** method is not advised when sample sizes are small or when the shape parameter(s) are less than 0.5, because highly skewed data increases the risk of parameter estimation instability. The possible inflation of type-I error when using parametric bootstrap analysis have been observed by many researchers in different settings; e.g. *Golzarri-Arroyo et.al.* [55]. Hence, we advise practitioners use caution when applying parametric bootstrappjng method in practice.

We expect the proposed methods have broad applicability to differential analysis in genomics studies and other applied fields. The proposed approaches for hypothesis testing and confidence interval estimation are easy to implement and their running time of these methods is quite feasible on standard computer platforms.

The R program is available at request from Dr. Yan at li.yan@roswellpark.org.

## Supporting information

S1 Table Real data example. The data used in Tables 4 and 5.
(XLSX)

S1 Appendix The characteristics of exponential-gamma (Exp-gamma) distribution. (PDF)

S2 Appendix Generalized pivots and generalized test variables. (PDF)
