# Supplementary material for: Statistical methods for comparing two independent exponential-gamma means with application to single cell protein data
Source: PLoS One. 2024 Dec 13;19(12):e0314705. doi: 10.1371/journal.pone.0314705 (PMC11643000; doi:10.1371/journal.pone.0314705)
Supplement: S1 Appendix — (PDF) [file pone.0314705.s002.pdf]

Appendix 1. The characteristics of exponential-gamma (Exp-gamma) distribution

Let $Y = \ln(X)$ stands for a random variable from Exp-gamma distribution, where $X \sim gamma(\alpha, \beta)$.

First consider the special case that $\beta = 1$. In this case, the probability density function of the X is

$$f_X(x) = \frac{1}{\Gamma(\alpha)} x^{\alpha-1} \exp[-x],$$

and the probability density function of Y is

$$f_Y(y) = \frac{1}{\Gamma(\alpha)} \exp[\alpha y - e^y].$$

The mean of Y equals to $\psi(\alpha) - \ln\beta$, and the variance can be calculated as follows:

$$\begin{aligned} Var(Y) &= E(Y^2) - E(Y)^2 \\ &= \frac{1}{\Gamma(\alpha)} \frac{d^2}{d\alpha^2} \int_{\Re} \exp(\alpha y - e^y) dy - E(Y)^2 \\ &= \frac{d^2}{d\alpha^2} \ln\Gamma(\alpha) \end{aligned}$$

Noting that $1/\beta$ acts as a scaling parameter on a gamma-distributed random variable,

$$X \sim Gamma(\alpha, 1) \Rightarrow \frac{1}{\beta} X \sim Gamma(\alpha, \beta),$$

and that a scaling parameter acts additively on the logarithmic expectation of a random variable,

$$\begin{aligned} & E[(\ln cX)^2] - E(\ln cX)^2 \\ = & E[(\ln X + \ln c)^2] - (E(\ln X) + \ln c)^2 \\ = & \frac{d^2}{d\alpha^2} \ln\Gamma(\alpha) = \psi^{(1)}(\alpha) \end{aligned}$$

Hence the variance of Y doe snot depend on β.

The moment generating function $M(t)$ of Y is

$$\begin{aligned} M(t) &= E[e^{tlnX}] = E[X^t] \\ &= \frac{\beta^\alpha}{\Gamma(\alpha)} \int_0^\infty x^{\alpha+t-1} e^{-\beta x} dx \end{aligned}$$

Noting that $f(x; \alpha + t, \beta) = \frac{x^{\alpha+t-1} e^{-\beta x} \beta^{\alpha+t}}{\Gamma(\alpha+t)}$, then

$$\begin{aligned} M(t) &= \frac{\beta^\alpha}{\Gamma(\alpha)} \frac{\Gamma(\alpha+t)}{\beta^{\alpha+t}} \frac{\beta^{\alpha+t}}{\Gamma(\alpha+t)} \int_0^\infty x^{\alpha+t-1} e^{-\beta x} dx \\ &= \frac{\beta^\alpha}{\Gamma(\alpha)} \frac{\Gamma(\alpha+t)}{\beta^{\alpha+t}} (F(\infty; \alpha+t, \beta) - F(0; \alpha+t, \beta)) \\ &= \frac{\Gamma(\alpha+t)}{\Gamma(\alpha)\beta^t} \end{aligned}$$

Hence the cumulant generating function is

$$\begin{aligned} K(t) &= lnM(t) \\ &= ln\Gamma(\alpha+t) - ln\Gamma(\alpha) - tln(\beta) \end{aligned}$$

and its mth order derivative is $K^{(m)}(0) = \psi^{(m-1)}(\alpha)$, $m \geq 2$. Therefore, the skewness ($skew$) and kurtosis ($kurt$) of Y can be easily obtained:

$$\begin{aligned} skew &= \frac{E[(Y - E[Y])^3]}{Var(Y)^{3/2}} = \frac{\psi^{(2)}(\alpha)}{[\psi^{(1)}(\alpha)]^{3/2}}, \\ kurt &= \frac{E[(Y - E[Y])^4]}{Var(Y)^{4/2}} = \frac{\psi^{(3)}(\alpha)}{[\psi^{(1)}(\alpha)]^{2}}, \end{aligned}$$

where $\psi^{(1)}()$, $\psi^{(2)}()$, and $\psi^{(3)}()$ are the second derivative, the third derivative, and the forth derivative of the log gamma function, respectively.
