# Supplementary material for: Statistical methods for comparing two independent exponential-gamma means with application to single cell protein data
Source: PLoS One. 2024 Dec 13;19(12):e0314705. doi: 10.1371/journal.pone.0314705 (PMC11643000; doi:10.1371/journal.pone.0314705)
Supplement: S2 Appendix — (PDF) [file pone.0314705.s003.pdf]

Appendix 2. Generalized pivots and generalized test variables

In the following, we will briefly review the concepts of generalized pivots and generalized test variables developed by Tsui and Weerahandi [40] and Weerahandi [41].

Suppose that a random sample, $\boldsymbol{Y} = (Y_1, \ldots, Y_n)^T$ from a distribution which depends on the parameter $\theta = (\psi, \boldsymbol{\nu})$ where ϕ is the parameter of interest and $\boldsymbol{\nu}^T$ is a vector of nuisance parameters. A generalized pivots $R(\boldsymbol{Y}; \boldsymbol{y}, \psi, \boldsymbol{\nu})$, where $\boldsymbol{y}$ is the observed value of $\boldsymbol{Y}$, for interval estimation has the following to properties [41]:

(1) $R(\boldsymbol{Y}; \boldsymbol{y}, \psi, \boldsymbol{\nu})$ has a distribution free of unknown parameters.
(2) The value of $R(\boldsymbol{y}; \boldsymbol{y}, \psi, \boldsymbol{\nu})$ is ψ.

Let that R_p be the $100p$th percentile of R. Then, $(R_{p/2}, R_{1-p/2})$ is the $100p\%$ two-sided generalized confidence interval for ψ.

Consider testing $H_0 : \phi = \psi_0$ vs. $H_0 : \phi > \psi_0$, where psi_0 is a specified quantity. A generalized test variable of the form $T(\boldsymbol{Y}; \boldsymbol{y}, \psi, \boldsymbol{\nu})$ satisfies the following conditions [40]:

(1) For fixed $\boldsymbol{y}$, the distribution of $T(\boldsymbol{Y}; \boldsymbol{y}, \psi, \boldsymbol{\nu})$ is free of $\boldsymbol{\nu}$.
(2) The value of $T(\boldsymbol{y}; \boldsymbol{y}, \psi, \boldsymbol{\nu})$ is free of unknown parameters.
(3) The fixed $\boldsymbol{y}$ and $\boldsymbol{\nu}$, and for all t, $P(T(\boldsymbol{Y}; \boldsymbol{y}, \psi, \boldsymbol{\nu}) > t)$ is either an increasing or a decreasing function of ψ.

A generalized extreme region is defined as $C = [T(\boldsymbol{Y}; \boldsymbol{y}, \psi, \boldsymbol{\nu}) > T(\boldsymbol{y}; \boldsymbol{y}, \psi, \boldsymbol{\nu})]$ if $T(\boldsymbol{Y}; \boldsymbol{y}, \psi, \boldsymbol{\nu})$ is stochastically increasing in ψ; otherwise, $C = [T(\boldsymbol{Y}; \boldsymbol{y}, \psi, \boldsymbol{\nu}) < T(\boldsymbol{y}; \boldsymbol{y}, \psi, \boldsymbol{\nu})]$. The generalized p-value is defined as $P(C \mid \psi_0)$.
